# The Assessment of Medical Device Software Supporting Health Care Services for Chronic Patients in a Tertiary Hospital: Overarching Study

**DOI:** 10.2196/40976

**Published:** 2023-01-04

**Authors:** Erik Baltaxe, Hsin Wen Hsieh, Josep Roca, Isaac Cano

**Affiliations:** 1 Institute of Pulmonary Medicine Chaim Sheba Medical Center Ramat Gan Israel; 2 Hospital Clinic de Barcelona Institut d’Investigacions Biomèdiques August Pi i Sunyer Universitat de Barcelona Barcelona Spain

**Keywords:** chronic patients, digital health, health technology assessment, implementation research, integrated care

## Abstract

**Background:**

Innovative digital health tools are increasingly being evaluated and, in some instances, integrated at scale into health systems. However, the applicability of assessment methodologies in real-life scenarios to demonstrate value generation and consequently foster sustainable adoption of digitally enabled health interventions has some bottlenecks.

**Objective:**

We aimed to build on the process of premarket assessment of 4 digital health interventions piloted at the Hospital Clinic de Barcelona (HCB), as well as on the analysis of current medical device software regulations and postmarket surveillance in the European Union and United States in order to generate recommendations and lessons learnt for the sustainable adoption of digitally enabled health interventions.

**Methods:**

Four digital health interventions involving prototypes were piloted at the HCB (studies 1-4). Cocreation and quality improvement methodologies were used to consolidate a pragmatic evaluation method to assess the perceived usability and satisfaction of end users (both patients and health care professionals) by means of the System Usability Scale and the Net Promoter Score, including general questions about satisfaction. Analyses of both medical software device regulations and postmarket surveillance in the European Union and United States (2017-2021) were performed. Finally, an overarching analysis on lessons learnt was conducted considering 4 domains (technical, clinical, usability, and cost), as well as differentiating among 3 different eHealth strategies (telehealth, integrated care, and digital therapeutics).

**Results:**

Among the participant stakeholders, the System Usability Scale score was consistently higher in patients (studies 1, 2, 3, and 4: 78, 67, 56, and 76, respectively) than in health professionals (studies 2, 3, and 4: 52, 43, and 54, respectively). In general, use of the supporting digital health tools was recommended more by patients (studies 1, 2, 3, and 4: Net Promoter Scores of −3%, 31%, −21%, and 31%, respectively) than by professionals (studies 2, 3, and 4: Net Promoter Scores of −67%, 1%, and −80%, respectively). The overarching analysis resulted in pragmatic recommendations for the digital health evaluation domains and the eHealth strategies considered.

**Conclusions:**

Lessons learnt on the digitalization of health resulted in practical recommendations that could contribute to future deployment experiences.

## Introduction

Over the past years, substantial progress has been made toward the adoption of digital technology for health or digital health [1]. Health programs using digital technology are increasingly being tested, evaluated, and, in some instances, integrated at scale into health information systems [2], considering its unique methodological challenges [3-6]. Investment in digital health requires evidence to support its value and expected benefits from the perspective of key stakeholders, that is, patients, professionals, health service providers, policy makes, and payers [7].

Assessment of digital health interventions has been conceptualized by a range of different evaluation frameworks [8,9]. A common factor in all of them is that digital health interventions should show benefit from all stakeholders’ perspectives during design and development [10], as well as after adoption. However, while such evaluation frameworks may serve as relevant guidelines, only recently, few of them have been extensively applied for the assessment of mature digital health tools [11-14]. Moreover, current medical device regulatory guidelines for digital health technologies [15-17] are, de facto, establishing their own evaluation constraints.

Common requirements are that digital health tools must be safe and must generate value to be successfully adopted in health care. Current medical device software (MDSW) regulations aim for successful links between privacy and information security, as well as for patient safety and clinical benefit [18,19]. It is of note that recent regulatory frames [14], aiming for fast-track assessment of digital applications, define a full set of requirements with respect to user friendliness, robustness, interoperability, and reimbursability.

The main objective of this research was to generate recommendations and to report lessons learnt from separate assessments of MDSW with the aim of bringing together the premarket experience from the cocreation of digital health interventions at the Hospital Clinic de Barcelona (HCB) during the period 2017-2019 [20] and the postmarket experience of MDSW after regulatory compliance in the European Union and the United States through MDSW recalls during the past 5 years (2017-2021). Due to the proliferation of innovation projects involving digital health interventions, there is a clear need for concerted efforts to harmonize and learn from both premarket piloting and postmarket surveillance experiences to foster applicability and standardization of the assessment of digital health tools in real-life settings.

It is of note that the premarket co-design experiences from the HCB [20] benefited from the combined input of all the stakeholders, including end users, aiming to prevent failures when reaching the market, and that the HCB has a dual role as a university center and as a driver of large community-based integrated care in the city of Barcelona (Área Integral de Salud de Barcelona Esquerra [AISBE]; 520,000 citizens) [21,22], falling within the activities of the Catalan Open Innovation Hub on Digitally Enabled Integrated Care Services, which is 1 of the 4 original EU Good Practices in the European Joint Action JADECARE [23].

## Methods

### Premarket Analysis of the Four Digital Health Prototypes Piloted at the HCB

Cocreation and quality improvement methodologies reported previously [20] were used to consolidate a pragmatic assessment protocol that was applied in the 4 digital health prototypes supporting the interventions (studies 1-4) described below.

Study 1 involved home-based noninvasive ventilation (NIV) of patients with hypercapnic respiratory failure. The study addressed enhanced management of chronic patients requiring specialized respiratory care for home-based NIV by means of a mobile app for patient self-management [24].

Study 2 involved prehabilitation of high-risk patients undergoing major abdominal surgery. The study assessed the potential of the supporting digital health tool, PREHAB [25], to enhance collaborative work among health professionals and patients using a mobile app for self-management at the community level.

The third cluster involved community-based care of frail chronic patients. This cluster of digital health interventions included 2 studies addressing specific objectives: (1) Study 3 assessed an adaptive case management platform [26] for community-based care of chronic patients; and (2) Study 4 investigated the potential of a secure communication platform, prototyped during 2019, for enhanced management of frail chronic patients [27] (Table 1). The details of the digital health interventions are provided in Multimedia Appendix 1.

**Table 1:** Details of the 4 digital health interventions (studies 1-4) piloted at the Hospital Clinic de Barcelona.

Study	Design	Inclusion and exclusion criteria	Digital health intervention
1	Single-blinded single-center RCT^a^ with 2 parallel arms (1:1 ratio): (1) control group (n=34) and (2) digital health intervention during a period of 3 months (n=33).	Inclusion criteria: Adult patients under home-based NIV^b^ at the HCB^c^ and having a mobile phone or tablet in the intervention group. Exclusion criteria: Patients with severe psychiatric or neurological diseases, as well as those hospitalized at the time of assessment.	MyPathway app (TRL^d^ 5) [28] was used for bidirectional interaction with the research team. It consisted of positive feedback or reinforcement messages in response to the number of hours of NIV use reported by the patient daily. Moreover, general advice on specific NIV clinical problems was automatically provided by the app according to the patients’ weekly input.
2	Prospective cohort study with 16 candidates for the prehabilitation service at the HCB.	Inclusion criteria: Candidates of major elective surgery in at least 4 weeks, age >70 years, an ASA^e^ score of III/IV, and access to a mobile phone or tablet with internet connection. Exclusion criteria: Physical or psychological problems affecting use and not having a career.	A digital health tool (TRL 6) [25] was used by health care professionals to prescribe and monitor tasks for patient self-management supported by an app, including physical activity goals, nutritional advice, mindfulness exercises, and predefined data collection instruments for patient-reported outcomes and experience.
3	Prospective cohort study with 20 clinically stable chronic patients recruited in 1 primary care unit from AISBE^f^ and followed-up for a period of 1 month.	Inclusion criteria: Acceptance to participate and having an appropriate smartphone or tablet. Exclusion criteria: Physical or psychological problems precluding the use of the app and not having a career.	Patients were given access to the platform (TRL 5) through their smartphones, and a pedometer was provided to track adherence to a personalized daily physical activity prescription, with remote support from a case manager.
4	Cluster RCT by primary care teams from AISBE, with an intervention (n=31) to control ratio of 2:1 and follow-up for a period of 3 months.	Inclusion criteria: Acceptance to participate and having an appropriate smartphone or tablet Exclusion criteria: Physical or psychological problems precluding the use of the digital tool and not having a career.	A case manager nurse used the Health Circuit (TRL 5) communication channel to trigger bilateral or group conversations, including health information exchange among specialized care, social care professionals, and community-based services, to agree on a goal-oriented and personalized health plan to manage both expected and unexpected events communicated by study participants [27].

^a^RCT: randomized clinical trial.

^b^NIV: noninvasive ventilation.

^c^HCB: Hospital Clinic de Barcelona

^d^TRL: technology readiness level.

^e^ASA: American Society of Anesthesiologists.

^f^AISBE: Área Integral de Salud de Barcelona Esquerra.

The premarket assessment of the 4 digital health interventions (Table 1) piloted during the period 2017-2019 primarily focused on assessment of technical robustness and usability. The former was assessed with a technical log book on the cloud that was updated daily with technical issues and suggestions for improvement from all study participants. The end users’ perceived usability and satisfaction were assessed by means of the System Usability Scale (SUS) [29] and Net Promoter Score (NPS) [30], alongside general questions about satisfaction.

Besides the technical and usability assessments mentioned above, compliance with other operational aspects, such as privacy and security, interoperability, transferability, and value generation of the accompanying integrated care services, was part of an overall evaluation framework [31], but the premarket analysis did not systematically analyze this. It is worth mentioning that digital support for the 4 digital health interventions (Table 1) was designed to operate on top of existing hospital information systems to minimize the need for ad-hoc integration via standard application programming interfaces.

When writing this manuscript, we adhered to the Consolidated Criteria for Reporting Qualitative Research (COREQ) [32]. All information retrieved from the technical and usability assessments of the 4 digital health interventions (including the end users’ interviews) was processed according to the protocol-specific ethics statement mentioned in the Ethics Approval and Consent section.

### MDSW Regulations and Postmarket Surveillance Analysis

In recent years, digital health technology has developed rapidly in the market as software-only novel therapies or has been embedded into medical devices or clinical workflows as a companion MDSW device in the market. MDSW is defined, under EU Regulation (EU) 2017/745-MDR [16], as a software, used alone or in combination, that is intended by its manufacturer as a medical device for human beings for a specific medical purpose: diagnosis, prevention, investigation, monitoring, prediction, treatment, alleviation, prognosis, and prediction.

The analysis of the European MDSW regulatory frame was focused on the EU Regulation 2017/745-MDR [16] and the fast-track process generated by Germany’s Federal Institute for Drugs and Medical Devices, known as the “BfArM” guidelines for the evaluation of digital health applications (DiGA) [14]. Likewise, for the United States, we considered the FDA 21 CFR Part 820 [15]. Moreover, the EU General Data Protection Regulation (GDPR) [18] and its American counterpart, the US Health Insurance Portability and Accountability Act (HIPAA) [19], were also considered in the analysis.

Within the postmarket surveillance analysis at the EU level, we collected the numbers of devices that had been recalled, irrespective of their risk level and the product end user, over 5 years (January 1, 2017, to December 31, 2021) from the German BfArM website [33]. The results included malfunctioning software that may result in a severe adverse event, device deficiency, incident, or serious incident.

For the postmarket surveillance analysis in the United States, we collected data from the following 2 public databases: Manufacturer and User Facility Device Experience (MAUDE) database [34] and MEDSUN Reports [35]. Considered MDSW recalls included software failures in terms of security flaws, privacy risks, internal controls, technical controls, physical controls, and implementation.

### Overarching Analysis

As a result of the experience-based cocreation process [20], 4 domains of digital health system validation (Table 2) and 3 eHealth contextual strategies (Table 3) were considered essential for the assessment of digital health tools. Therefore, they were used to guide a thematic analysis on the assessment results of the 4 digital health interventions, by the author EB, as well as on the overview of the MDSW current regulations and the analysis of MDSW recalls of the past 5 years (2017-2021) in the European Union and the United States, by the author HWH.

Then, the author EB discussed the findings with the participants of each digital health intervention, the final users of the supporting digital health tools, and all other coauthors. For each of the 4 digital health interventions, the evaluation results were judged by the author EB according to the contextual eHealth strategy, defined by the role of the digital tools in the health care service.

Finally, we evaluated the thematic analysis results to generate recommendations for assessment of the sustained adoption of future digital health interventions.

**Table 2:** The domains considered essential for the validation of digital health systems [20].

Domain and component		Description
**Technical**		
	Robustness	Testing of performance when compared to a technical gold standard
	Privacy and security	Testing of privacy and security requirements
	Interoperability	Testing of interoperability requirements
	Transferability	Potential to adapt to other services and implementation scenarios
	Smartness	Testing of innovative features powered by artificial intelligence
**Clinical**		
	Safety	Critical appraisal of technology impact on patient safety outcomes
	Medical benefit	Evidence of positive health care effects
**Usability**		
	Ease of use	Whether digital health systems can be used as intended by users
	Feasibility	Whether digital health systems work as intended in each context
**Cost**		
	Value generation	Anticipated cost impact on the clinical outcome of interest
	Affordability	If the costs of digital health systems can be made affordable

**Table 3:** The eHealth contexts considered essential for the validation of digital health systems.

eHealth strategy	Description
Telehealth	Digital support to well-established service workflows to enhance health care efficiencies. Typically, clinical evidence is not required. Not a medical device.
Integrated care	Digital support to enable innovative service workflows with a care continuum approach. Clinical evidence is required. Requires regulatory clearance or approval.
Digital therapeutics	Medical device software–driven therapeutic intervention for prevention, management, or treatment. Evidence and regulatory approval are required.

### Ethics Approval and Consent

Letters of medical ethics approval of the Ethics Committee for Medical Research of the HCB and signed informed consent forms were obtained for the 4 studies (study 1, HCB/2019/0510; study 2, HCB/2016/0883; study 3, HCB/2018/0803; and study 4, HCB/2018/0805).

## Results

### Premarket Analysis of the Pilots

A total of 99 chronic patients and 9 health care professionals were assessed during the interaction and cocreation process of the 4 different digital health interventions piloted at the HCB (studies 1-4). Assessment results of the technical and usability performances are summarized in Table 4. See Multimedia Appendix 2 for further details.

**Table 4:** Usability performance and summary of the technical log book reported by patients and professionals with respect to the digital health tools supporting the 4 digital health interventions piloted at the Hospital Clinic de Barcelona.

Study	Patients’ experience				Professionals’ experience				Technical log book^a^	
	n	NPS^b^	SUS^c^ score	n		NPS^b^	SUS^c^ score			
1	33	−3%	78	1		N/A^d^	N/A	Recurrent login with a username and a password that are easy to forget (patients).		
2	16	31%	67	2		−67%	52	Technology bugs (health professionals) and system enforcement for a random password after reset (patients).		
3	19	−21%	56	1		1%	43	Problems connecting the pedometer via Bluetooth with some Android smartphones (patients).		
4	31	31%	76	5		−80%	54	Lack of robustness of the multimedia communication channel with some Android smartphones (health professionals).		

^a^Main reported issues from patients or health professionals.

^b^The Net Promoter Score (NPS) is a known questionnaire used to assess satisfaction with a product, which includes a key question: “How likely is it that you would recommend our system to a family member or friend?” Patients can give an answer ranging from 0 (“not at all likely”) to 10 (“extremely likely”). Individuals scoring 9 or 10 are called “promoters,” individuals scoring 7 or 8 are called “passives” (or neutrals), and individuals scoring 0 to 6 are called “detractors.” The NPS is computed as percent promoters − percent detractors, and ranges from −100% to 100%.

^c^The System Usability Scale (SUS) was developed by John Brooke in 1986 and consists of a 10-item questionnaire scored on a 5-point Likert scale from 0 (strongly disagree) to 5 (strongly agree). The overall score is calculated from the sum of all item scores multiplied by 2.5 and can range from 0 to 100. A system or product that receives a score of 68 or above is considered to have good usability.

^d^N/A: not applicable.

### Study 1: Home-Based NIV of Patients With Hypercapnic Respiratory Failure

Most (20/27, 74%) reported incidences by end users had to do with the need to login with a username and a password that were easy to forget, which precluded the ease of use. However, on a Likert scale from 1 (very bad) to 10 (very good), the general impression of patients was scored 7.5, user friendliness was scored 8.2, and the ability to use the app without assistance was scored 8.5. This was in line with the mean patient usability score (78 out of 100).

### Study 2: Prehabilitation of High-Risk Patients Undergoing Major Abdominal Surgery

Fifty percent (5/10) of incidences were due to comfortability and accessibility (the system forced the use of a random password when the password was reset) and 30% (3/10) were due to technology robustness. The remaining 20% (2/10) of incidences were due to various factors. As in study 1, the general impression and user friendliness was scored 8 (out of 10) and the ability to use the app without assistance was scored 7.5 (out of 10). In contrast, the patient usability score had a mean value of 67, which is considered an average usability grading. With respect to the experience of professionals, a neutral experience using the web backend for professionals was reported, with an overall satisfaction score of 5 (out of 10) and a mean SUS score of 52.

### Study 3: Community-Based Care of Frail Chronic Patients With the CONNECARE Platform

Most (4/7, 57%) observations during the pilot were due to lack of robustness of the Bluetooth connection with the pedometer. Reported observations regarding motivation, reliability, comfortability, and accessibility reached 14% (1/7) each. In general, patients had a slightly positive experience (6/10) using the system, but its usability was graded low (SUS score of 56). In case of professionals, perceived usability was graded lower (SUS score of 43); thus, the professionals involved would not recommend the CONNECARE system.

### Study 4: Community-Based Care of Frail Chronic Patients With the Health Circuit Prototype

High proportions of observations were due to usability (11/18, 61%), and comfortability and accessibility (6/18, 33%) issues, mostly due to lack of robustness of the multimedia communication channel. In general, most patients had a positive experience using the system, which was reinforced by the fact that the median overall satisfaction score was 7.8 (out of 10) and the mean patient usability score was 76. However, the NPS reported by professionals (n=5) was negative (−80%), but since the median overall satisfaction score was 5 and the perceived usability score was 54, we could consider that professionals had a neutral experience using the prototype.

### Comparability Analysis of EU and US MDSW Regulations

#### Data Protection

Both the EU and US regulatory frames (GDPR [18] and HIPAA [19], respectively) provide clear guidelines for manufacturers, health professionals, patients, and users in general, to assess how medical devices protect private information and security. The GDPR governs the use of and applies to all personal data from an individual person who is in an EU country at the time the data are collected, while HIPAA has a much narrower scope and only applies to protected health information. However, both the regulations are established keeping in mind the public interest and security of sensitive information [36]. In addition, potential risk management assessment and cyber security are essential to consider the stage of design, development, clinical investigation, and postmarket surveillance. Since the primary focus is on data security, privacy, and integrity, all the measures necessary to comply with the regulations are broadly similar. Thus, MDSW that are already GDPR or HIPAA compliant will have in place most of the security measures required to protect data privacy.

#### Medical Devices

To ensure the safety and efficiency of medical devices while supporting innovation, the European Union and United States have established their own transparency route to internal markets and a procedure for verification and validation (Medical Device Regulation [MDR] [16] and Food and Drug Administration [FDA] [15], respectively). Medical device regulatory compliance under both the FDA and MDR is a complex path involving processes that need constant monitoring and maintenance. For example, the recent requirements of the MDR are much closer to those of the FDA in terms of (1) prerequisites for the conformity assessment; (2) a quality management system in-place compliant with ISO 13485; and (3) use of consensus standards that are relevant to the development and design of interoperable medical devices. With that said, there are some key differences. The FDA’s classification system is based upon 3 risk classes, while the EU MDR has 4 device categories and 5 risk-based classifications.

The risk classification assigned will determine the depth and amount of clinical data required under the MDR to get approval for the medical device, whereas under the FDA, Class I and some Class II devices do not require clinical testing, and only proof is required that the medical device is substantially equivalent to a legally marketed product.

#### Toward Digital Therapeutics

Perhaps the biggest challenge facing EU and US MDSW regulations is reimbursement. With evidence supporting the efficacy of digital therapeutics stacking up, more payers are coming round to the idea of MDSW reimbursement and the business case for offering it [37]. With it becoming ever clearer that MDSW, in general, and digital therapeutics, in particular, can play significant roles in the treatment of many conditions around the world, both the European Union and FDA are creating regulatory frameworks for the safety and efficacy of digital therapeutics. Germany launched a fast-track process for digital health applications (DiGA) [14], which is the first in the world for digital therapeutics reimbursement. DiGA is a pathway for doctors to prescribe digital therapeutics to publicly insured patients and receive reimbursement in much the same way as traditional treatment. This catapulted Germany to global leadership in digital therapeutics regulation. No other country has yet made prescription digital therapeutics so widely available to such a high percentage of the population. Beyond EU MDR standards, DiGA defined further requirements, such as interoperability, robustness, and ease of use, among others.

### Postmarket Surveillance Analysis

The review of the German regulatory framework (BfArM) retrieved a total of 556 postmarket events that fitted the research requirements focused on digital health tools (representing 13% of all events that included drugs, assays, and medical devices). Likewise, the review of the MAUDE and MEDSUN databases (United States) found a total of 114 software-related issues, representing 18% of all queried events. See Multimedia Appendix 3 for details.

The vast majority of reported issues were related with software problems, incorrect results, data mismatch, error codes, system unexpected shutdowns, incorrect procedures, and cybersecurity-related aspects, which folded into the *robustness* (620/665, 93.2%) and *privacy and security* (26/665, 3.9%) components of the technical domain mentioned in Table 2. Therefore, software technical defects were reported to have the highest potential risk of harming end users.

No issues were reported with respect to *transferability*, *smartness*, *safety*, or *medical benefit*, whereas very few issues were reported in relation to *interoperability* (2/665, 0.3%), *ease of use* (8/665, 1.2%), and *feasibility* (9/665, 1.3%).

### Overarching Analysis

The overarching analysis of the process of premarket assessment of the 4 digital health interventions piloted at the HCB, as well as the analysis of current MDSW regulations and postmarket surveillance in the European Union and United States provided a source of experience-based knowledge that is described below and summarized in Tables 5 and 6 in terms of recommendations for each of the 4 digital health evaluation domains (Table 2) and lessons learnt toward sustained adoption in the 3 eHealth contexts considered (Table 3).

**Table 5:** Recommendations for the assessment of medical device software for sustained adoption of future digital health interventions.

Domain	Recommendations
Technical	A high technology readiness level is key for sustained adoption of MDSW^a^. Data privacy, security, and interoperability need to be addressed for regulatory compliance. MDSW should evolve to support collaborative work.
Clinical	A dedicated change management team is required for integrated care eHealth strategies. A unified evaluation protocol facilitates comparability among digital health interventions. Key performance indicators need to be adopted for continuous assessment.
Usability	Cocreation facilitates design while minimizing the need for user training and enhances adoption. Cognitive behavioral therapy techniques enhance user adherence to digital health applications.
Cost	Evidence on health care value generation of digital health interventions precedes cost containment. Bundle payment approaches based on service performance are advised.

^a^MDSW: medical device software.

**Table 6:** Lessons learnt for the assessment of medical device software for sustained adoption of future digital health interventions.

eHealth strategy	Lessons learnt
Telehealth	Digital health tools that engage consumers for lifestyle, wellness, and health-related purposes, which typically do not require regulatory oversight, do not ensure value generation.
Integrated care	Evidence-based MDSW^a^ that allow all stakeholders in the care continuum to collaborate and to access, share, aggregate, and visualize meaningful data daily, are expected to contribute the most to health care efficiency generation.
Digital therapeutics	Digital therapeutics that win public reimbursement must have solid proof of their efficacy/effectiveness. A market strategy or a MDSW regulation that helps build that proof is therefore essential. Real usage data for digital therapeutics and associated evaluations should determine national health coverage.

^a^MDSW: medical device software.

#### Technical Domain

Optimization of health care value generation and sustainability of the digitally enabled integrated care services explored in the 4 pilot studies were limited by the lack of technical robustness of the prototypes tested during the period 2017-2019, with technology readiness levels [38] within the interval 5-6.

It is of note that data privacy, information security, and data standardization are essential for enabling interoperability with health information systems from different providers or health information exchange platforms across providers within a geographical area. However, many privacy and security features are known to reduce user satisfaction.

In terms of interoperability and transferability, MDSW should evolve to support collaborative work among stakeholders across community and hospital services (ie, vertical and horizontal integration), using shared care plans that incorporate patient goals, which will foster the digital transformation of health care within a care continuum scenario.

#### Clinical Domain

Overall, digital support should be embedded into properly defined health care service workflows, particularly relevant for integrated care and to some extent digital therapeutic eHealth contexts. Moreover, implementation of adaptive case management for the management of care pathways is highly advisable to face the challenge of unexpected events within well-defined care paths. Telehealth tools not embedded into properly defined health care service workflows, focused on engaging consumers for lifestyle, wellness, or any other health-related purposes, which typically do not require regulatory oversight, do not ensure value generation.

The implementation of digitally enabled integrated care is disruptive and requires transformational change at all levels of an organization. This requires careful and solid strategic planning considering all the obstacles that may be encountered, as well as developing incentives and ongoing change management with a dedicated change management team. Pragmatic application of the same evaluation protocol is highly recommended to facilitate comparability among deployment experiences and to identify key performance indicators for long-term follow-up quality assessment of the service, beyond the initial deployment. In this regard, the use of profiled dashboards could be an efficient strategy for the assessment of cost-effectiveness in real-life settings, especially for MDSW using eHealth strategies that require evidence of efficacy and effectiveness (ie, integrated care and digital therapeutics).

#### Usability Domain

Flexible adoption of patient-centered cocreation methodologies during the premarket studies was useful to identify factors that generate bottlenecks, facilitating design and adoption of timely action plans. In general, cocreation efforts represented a success factor in terms of perceived usability by patients, but generated high expectations by health care professionals that were not met due to lack of technical robustness of the prototypes tested, which was a negative factor in terms of perceived usability. Efforts must be devoted toward the development of digital health applications that support patient empowerment for self-management with cognitive behavioral therapy to foster the long-term effectiveness of digitally enabled interventions.

#### Cost Domain

Digital transformation of health care must be based on cost containment. Operational costs of innovative, digitally supported, integrated care services are expected to decrease, so transitional costs should be covered by savings generated through decreases in operational costs. To this end, bundle payment approaches based on service performance are advised. Evidence-based MDSW embedded into properly defined health care service workflows with an integrated care approach are expected to contribute the most to health care efficiency generation. MDSW delivering a therapeutic intervention must have solid proof of efficacy in controlled clinical trials, but real usage data should be used to monitor cost-effectiveness in a real-world setting and ultimately determine national health coverage.

#### eHealth Strategies

Digital health tools have become integral to the prevention, diagnosis, treatment, and management of health and diseases. Clinicians use digital health tools to gain insights into patient outcomes, conduct telehealth visits, treat aspects of diseases otherwise unaddressed by traditional medications, and, ultimately, ensure health care efficiency generation. It is crucial to describe the landscape of available digital health tools in addition to the level of clinical evidence and regulatory oversight that correlates with each eHealth category in this quickly evolving industry. End users, clinicians, and payers should understand the difference between the purpose and function of various MDSW, since this differentiation determines the risk level assumed for clinical evidence generation alongside the technical requirements for regulatory oversight.

## Discussion

### Summary of the Results

The study aimed to update health professionals on the current landscape of MDSW for enhanced management of chronic patients. The research generated recommendations on target evaluation domains and eHealth categories through an overarching analysis of 3 sources of information: (1) premarket evaluation of 4 pilots carried out at the HCB, using ongoing technological developments; (2) assessment of the regulatory frames of MDSW in the United States and Europe; and (3) postmarket surveillance reporting from the same 2 areas of the world.

Evaluation results of the 4 digital health interventions piloted at the HCB showed that patients tended to score higher than professionals in terms of the experience with supporting digital health tools, and in general, they would recommend the use of supporting digital health tools. This can be partly explained by the fact that the technology readiness level of the assessed digital health interventions was rather low at the precommercial stage, which most likely had a negative impact on the perceived usability by health care professionals who had to lead with technical issues at the same time than with the inherent complexities of case management. Moreover, the lack of integration with existing hospital information systems influenced the poor results with respect to the experience of health professionals. The 4 premarket digital health interventions were not considered mature for integration with existing health information systems, and in general, hospital information technology departments tend to reject integration of noncommercial digital health tools, which precludes usability, especially among health professionals who are not strongly motivated.

As mentioned above, EU and US MDSW regulations include premarket MDSW assessment with respect to clinical data, product information, performance testing, labeling, benefit-risk assessments, residual risks, etc. However, MDSW manufacturers should also plan, establish, document, implement, maintain, and update a postmarket surveillance system in a manner that is proportionate to the risk class and appropriate for the type of device. This system should be an integral part of the manufacturer’s quality management system and should be notified to the corresponding regulatory body.

Overall, the postmarket surveillance review of both German and US regulatory frameworks confirmed the crucial role of software verification and validation, the voluntary testing of cybersecurity, and the need for testing user interfaces.

### Strengths and Weaknesses

The inherent heterogeneity of the 4 digital health interventions considered in this study represents both a strength, because it reinforces transferability of the assessment approach, and a limitation, because it precludes comparability of the results among the 4 study protocols. However, the cocreation process and the application of the same structured evaluation protocol over the 4 digital health interventions contributed to the evolution of the mindset of health professionals toward the use of digital health tools. Specifically, the participation of all stakeholders in the overarching analysis concluded with the generation of a set of general recommendations for adoption in routine clinical practice.

The evaluation protocol focused on technical and usability performance because the primary objective of the 4 digital health interventions piloted at the HCB was to demonstrate the feasibility of the approach. If large-scale deployment is the primary aim, other functional, technical, and ethical aspects of the supporting digital health tools will need to be assessed.

The 4 digital health interventions were piloted within the context of research and innovation projects. This represents a clear advantage for stimulating cocreation of the supporting digital health tools, but establishes the threshold of required technological maturity at the prototype level and limits the transferability of the results beyond the boundaries of pilot settings.

Considered MDSW regulations in the European Union and United States neglect the tools and general requirements of other countries or regions of the world. Although similar premarket approval applications and postmarket surveillance tools are being put in place in recent years [11-14], they do not apply to the full range of technology readiness levels, that is, they are intended to be used to evaluate mature technology. In this respect, development and production phases of digital health tools (focus of the 4 digital health interventions assessed in this study) may help to generate more mature and robust digital health tools that are ready to be assessed by corresponding health technology assessment frameworks [11-14].

### Toward the Adoption of MDSW

Technical performance and usability are arguably among the most important considerations with patient-oriented mobile and digital-based solutions [39,40].

The overarching analysis showed a clear link between premarket assessment and postmarket surveillance in terms of technical failures hindering stakeholder adoption, regardless of usability and acceptability success. Such technical failures can be overcome by generating not only robust and secure products, but also online open access databases (the likes of clinical registries for single diseases) where basic MDSW approval information, medical specialty, and algorithm details can be publicly shared, thus enhancing transparency and collaborative work. Two recent studies [41,42] explored this concept when applied to artificial intelligence MDSW.

Moreover, digital health apps must be easy to use for their intended purpose, require minimal effort to complete tasks, have minimal data entry burden, and allow the user to control preferences when appropriate (eg, notifications). Since systems can be designed for users with disabilities (eg, impaired vision, motor deficits, and cognitive dysfunction), design considerations must ensure that accessibility compliance reflects the target user audience and different potential users, including family members and caretakers. Moreover, to maximize acceptability, digital health solutions require input from clinicians.

Developing digital health tools not only implies technological robustness and usability, but also guarantees data privacy. It requires thinking about how the newly collected data will need to be shared with health care professionals and whether the intended use of the technology ethically makes sense. During the long process of creating and validating a digital health application (starting with an idea, followed by its implementation and dissemination in different application markets), many stages must be achieved, and each one has its own particularities and methodologies.

Accordingly, a redefinition of the digital health ambit is needed. While some evaluation models in terms of the maturity of digital health interventions have been proposed [43], they are either very “technology specific” or “hospital oriented.” Moreover, as acknowledged previously [43], none of the identified models can be used as an overarching tool to encompass the wide range of digital tools used in a complex context such as integrated care. Moreover, they highlight the lack of a holistic approach to identify influencing factors.

The maturity grading criteria for digital health explored in this study cover a wide scope of tools and policies that correspond to the abovementioned new model of the comprehensive understanding of digital medicine. Recently, a review was conducted on the use of digital technologies in health care [44], and not surprisingly, it proposed an approach similar to the one proposed in the 4 domains and 3 eHealth contexts (Tables 2 and 3) to assess the different elements of MDSW adoption.

### Conclusions

Usability performance was consistently perceived higher by patients than by health care professionals. This can be partly explained by the fact that the technology readiness level of the supporting digital health tools was within the interval 5-6, and health care professionals had to lead with technical issues at the same time than with the inherent complexities of case management.

However, the active participation of health care professionals in the co-design and application of the evaluation protocol contributed to the evolution of the mindset of the health professionals toward the use of digital health tools in routine clinical practice.

The overarching analysis resulted in lessons learnt and recommendations that could contribute to the large-scale adoption of digital health tools.

## Appendix

Multimedia Appendix 1 Details of the digital health interventions.

Multimedia Appendix 2 Details of the premarket analysis of the 4 pilot studies.

Multimedia Appendix 3 Details of the postmarket surveillance analysis.

## Abbreviations

BfArM: Federal Institute for Drugs and Medical Devices
FDA: Food and Drug Administration
GDPR: General Data Protection Regulation
HCB: Hospital Clinic de Barcelona
HIPAA: Health Insurance Portability and Accountability Act
MAUDE: Manufacturer and User Facility Device Experience
MDR: Medical Device Regulation
MDSW: medical device software
NIV: noninvasive ventilation
NPS: Net Promoter Score
SUS: System Usability Scale
