# Supplementary material for: The Assessment of Medical Device Software Supporting Health Care Services for Chronic Patients in a Tertiary Hospital: Overarching Study
Source: J Med Internet Res. 2023 Jan 4;25:e40976. doi: 10.2196/40976 (PMC9873251; doi:10.2196/40976)
Supplement: Multimedia Appendix 1 [file jmir_v25i1e40976_app1.docx]

# **Multimedia Appendix 1.** Details of the digital health interventions.

# Study I – Digital support to Home-based non-invasive ventilation (NIV) of patients with hypercapnic respiratory failure

**Objective:** Customize MyPathway® for Home-based non-invasive ventilation (NIV) at HCB (Study I) and integrate MyPathway with the health information systems of Hospital Clínic (with a HL7-FHIR integration middleware).

As depicted in **Figure S1**, the NIV service considered as key supporting technologies a telehealth platform to enhance collaborative work among health professionals and patients themselves using a personal health system for patient self-management at community level with remote capture of patient reported outcomes (PROMs) to answer study questionnaires, delivery of general educational material and personalized recommendations and monitoring of self-reported hours of use (daily). Most importantly, these key supporting technologies were required to be ready for integration with Hospital Clínic information systems (i.e. SAP) and the regional health information systems for a large scale development in the region (i.e., Catalonia).


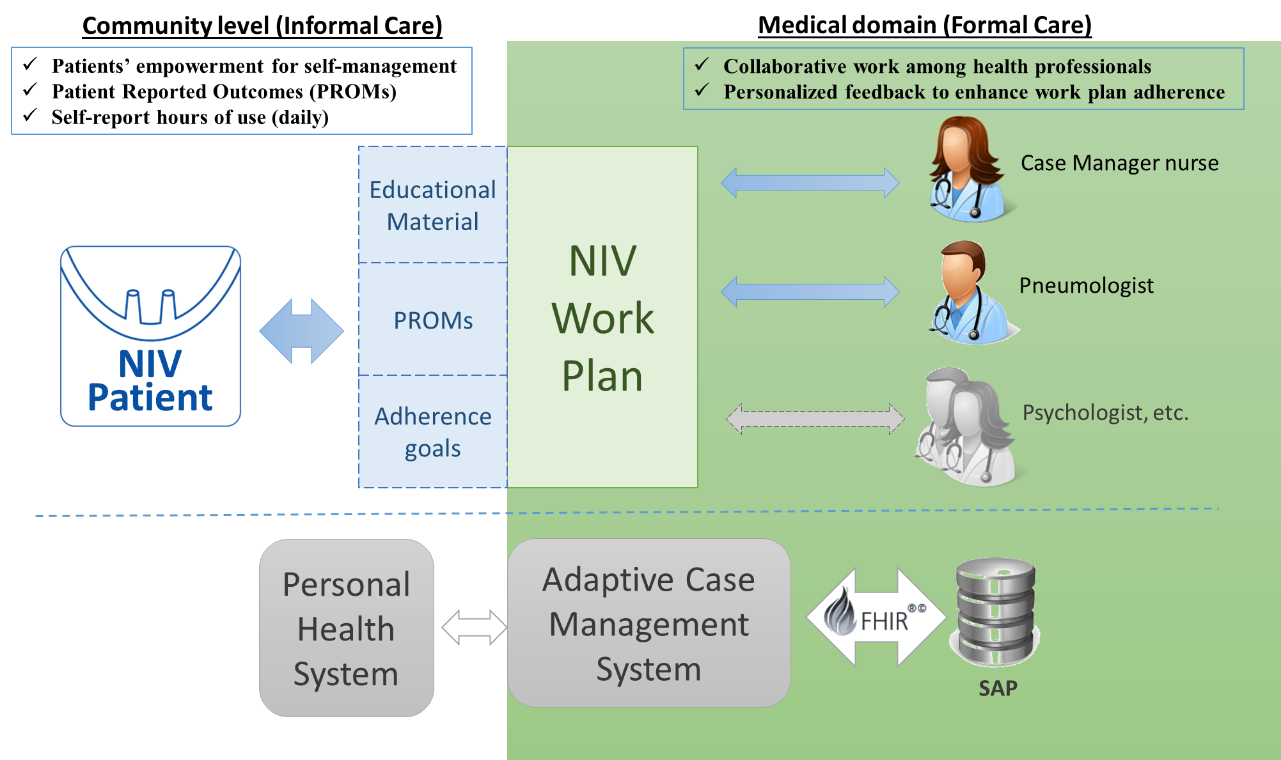


*Figure S1 - The figure shows two interoperable domains with technological elements providing support to NIV. On the left hand side, the Informal Care area considers the patient access to the NIV Personal Health System wherein she/he can answer study questionnaires (PROMs), report hours of daily use and have access to tailored educational information, as defined in the NIV work plan (centre of the figure). On the right hand side, the Formal Care domain includes the NIV team (Case manager nurse, pneumologist, Psychologist, etc.), with access to an adaptive case management system for work plan prescription, follow-up and coaching. The adaptive case management system supports execution of the patient work plan and provides a bridge of interoperability and collaborative tools among the patient (through the NIV personal health system), the NIV team and the electronic medical record (i.e. SAP in case of HCB).*

For the design, technical deployment and integration of the supporting technologies in a stepwise manner, the NIV service prioritised the implementation of the personal health system for patient self-management at community level. Specifically, a personal health system (MyPathway®) currently being adapted in hospitals in the UK (e.g., Sheffield Teaching Hospital) was adapted. MyPathway® is a secure, digital communications channel connecting patients to clinicians and services. It is a browser and app-based application co-designed and tested by users to make it user-friendly for both patients and clinicians to use on phones, tablets and PCs.

Although MyPathway® already allowed for automated PROMs capture, such as EQ-5D™, Oxford Hip Score and MSK-HQ, the NIV service had different requirements (**Table S1**), including the use of specific questionnaires to report predefined clinical problems: i) dry mouth, ii) red eyes, iii) mask noise, iv) mask leak sensation, v) diurnal somnolence and vi) weight gain. In addition, since a key requirement of the NIV service is the capacity to prescribe and remotely self-report daily use of NIV, MyPathway was extended to allow for manual prescription of daily NIV use goals.

Table S1 – Adaptation requirements of MyPathway to support the NIV service at HCB (Study I)

| **Feature** | **Description** |
| --- | --- |
| **Spanish and Catalan languages** | Hospital Clínic facilitates translation to Spanish and Catalan both for the clinician’s portal and the patient’s web/app |
| **Monitoring of patient’s daily use of NIV** | Patient-specific target daily use of NIV (i.e., number of daily hours) will be prescribed by healthcare professionals (number of target daily hours should be customisable dynamically and the prescription could be cancelled anytime.). Patients will receive the prescription in MyPathway timeline (in the form of a daily goal), to be manually answered by the patient. Based on self-reported hours of use of NIV, motivational feedback will prompt the patient to continue in the same line or try change his/her behaviour by identifying any of the specific problems mentioned below. |
| **PROMs** | Periodically (weekly or when the patient self-report less than 4 hours of daily use), MyPathway will use specific questionnaires to report predefined clinical problems: i) dry mouth, ii) red eyes, iii) mask noise, iv) mask leak sensation, v) diurnal somnolence and vi) weight gain. |
| **Integration with hospital information systems** | Patient referral to the NIV program will trigger the creation of a new user in the clinician’s portal and will send the invitation to the patient for registering to MyPathway. Acceptance of the invitation will trigger the allocation of the on-boarding material to the patient timeline. |

**Figure S2** below illustrates with screenshots the main functionality of the NIV personal health system.


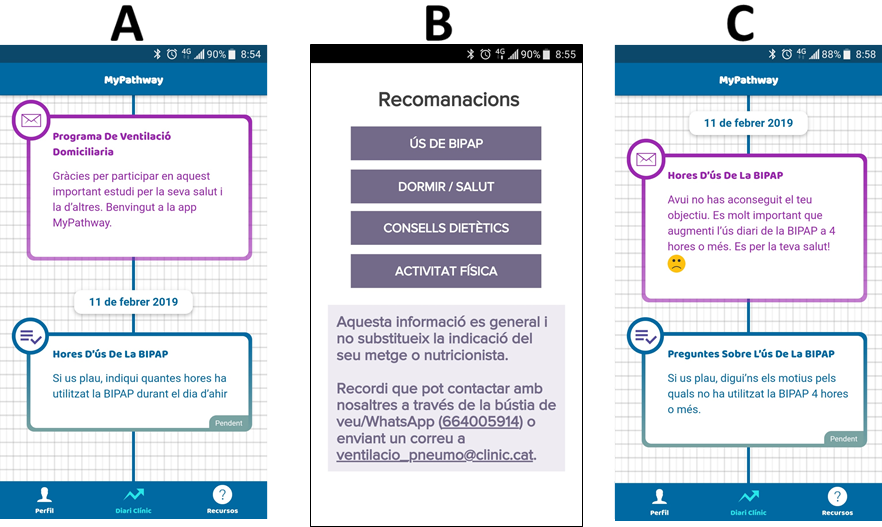


*Figure S2 – Look-and-feel of main functionalities of NIV personal health system: Welcome message (Panel A – purple timeline message) with link to on-boarding material and daily NIV use goal (Panel A – blue timeline message), educational material (Panel B), and tracking of goal progress with motivational feedback and self-administered PROMs (Panel C –blue timeline messages).*

Finally, MyPathway® was ready to be integrated with SAP via ORM messaging triggered by the eventual inclusion of a patient in the NIV program. The ORM message would be securely bypassed between SAP and MyPathway with a Fast Healthcare Interoperable Resource platform (HAPI FHIR) deployed in the intranet of hospital information systems. Such bypass consists on automatically sending an invitation letter to the e-mail of the patient (if already informed in the Hospital SAP) with instructions on how to access the browser and app-based version of the NIV personal health system and how to setup, for the first time, their password.

# Study II – Prehabilitation of high-risk patients undergoing major abdominal surgery

**Objective:** Customize the PREHAB system from EIT Health supported project PAPRIKA^[[1]](#footnote-1)^ for the prehabilitation unit on Barcelona (Study II) and integrate it with the health information systems of Hospital Clínic (with a HL7-FHIR integration middleware).

As depicted in **Figure S3**, the PREHAB service considered as key supporting technologies an adaptive case management platform to enhance collaborative work among health professionals and patients themselves using a personal health system for patient self-management at community level with off-line remote capture of patient reported outcomes (PROMs) and monitoring of daily physical activity (PA). Most importantly, these key supporting technologies were required to be integrated with Hospital Clínic information systems (i.e. SAP) and the regional health information systems for a large scale development in the region (i.e., Catalonia).


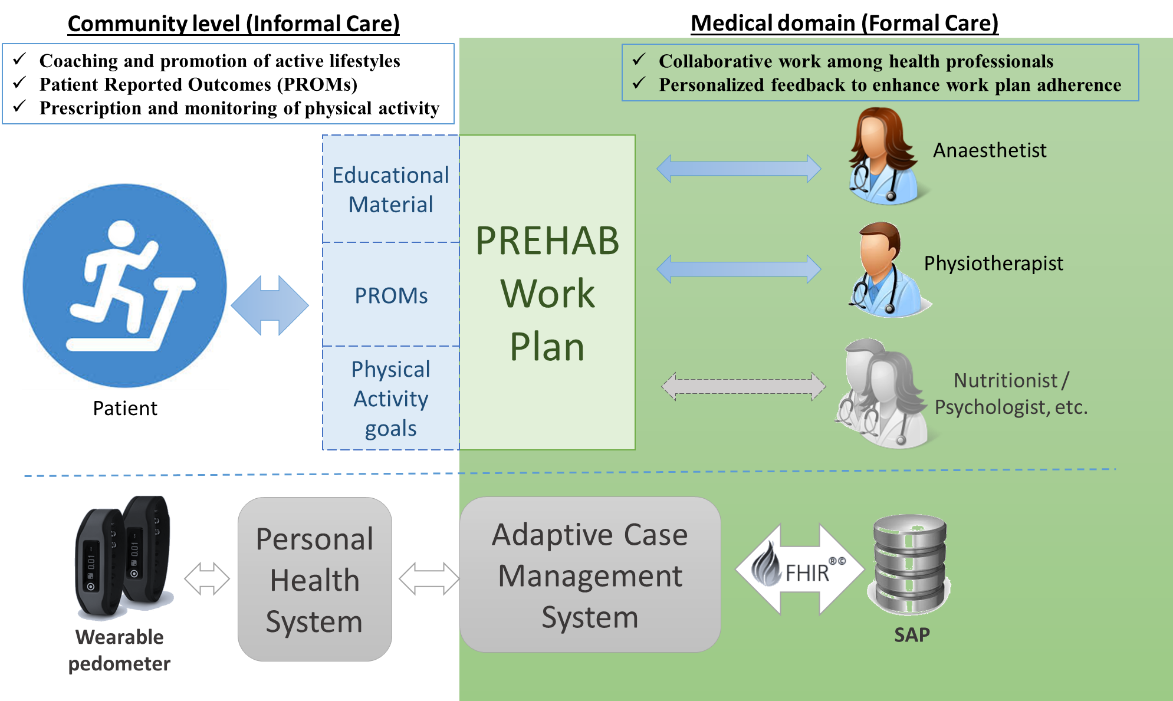


*Figure S3 - The figure shows two interoperable domains with technological elements providing support to the PREHAB services promoting active lifestyles as part of the action plan of the patient. On the left-hand side, the Informal Care area considers the patient access to the PREHAB Personal Health System wherein she/he can answer questionnaires (PROMs), perform physical activity monitoring through wearable pedometers and have access to tailored educational information, as defined in the PREHAB work plan (centre of the figure). On the right-hand side, the Formal Care domain includes the PREHAB team (Anaesthetist, physiotherapist, Nutritionist, Psychologist, etc.), with access to an adaptive case management system for work plan prescription, follow-up and coaching. The adaptive case management system supports execution of the patient work plan and provides a bridge of interoperability and collaborative tools among the patient (through the PREHAB personal health system), the PREHAB team and the electronic medical record (i.e. SAP in case of Hospital Clínic).*

A key requirement of the PREHAB service (**Table S2**) is the capacity to prescribe and remotely monitor patients’ daily PA (i.e., target number of daily steps) and its off-line remote monitoring with wearable pedometers. In a first phase, FitBit® pedometers were integrated so that tracked number of steps were collected from the FitBit® cloud. However, this required the persistent background execution of the FitBit® app for continuous synchronization between the pedometer and the FitBit® cloud, as well as the need for a FitBit® account, which introduced too much complexity to end-users. For this reason, a second phase is directly (i.e., API-based) integrating LifeVit® pedometers with the PREHAB personal health system using Bluetooth connectivity, removing the need for synchronization with third party cloud services.

*Table S2 – Adaptation requirements for PREHAB system to support the prehabilitation service on Hospital Clínic of Barcelona (Study II)*

| **Feature** | **Description** |
| --- | --- |
| **Spanish and Catalan languages** | Hospital Clínic facilitates translation to Spanish and Catalan both for the clinician’s portal and the patient’s web/app |
| **Monitoring of patient’s physical activity** | patient-specific target daily physical activity (i.e., number of daily steps) will be prescribed by healthcare professionals (number of target daily steps should be customisable dynamically and the prescription could be cancelled anytime.). Patients will receive physical activity prescriptions in MyPathway timeline (in the form of a daily goal), which can be manually answered or chosen to be directly linked via Bluetooth (requires integration of SDK) with a LiveVit pedometer (AT-250/AT-260) for automatic collection of daily steps. Patients will receive daily and weekly feedback (rewards, encroaching messages) with respect to the adherence to the physical activity goals. |
| **PROMs** | Spanish validated versions of the following questionnaires should be available for allocation at patient discharge:   - **YALE** - **HAD** - Patient Satisfaction questionnaire of the prehabilitation unit |
| **Integration with hospital information systems** | Patient referral to the prehabilitation program will trigger the creation of a new user in the clinician’s portal and will send the invitation to the patient for registering to PREHAB. Acceptance of the invitation will trigger the allocation of the on-boarding material (Introductory video of the prehabilitation unit and a pdf document with basic information of the prehabilitation program) to the patient timeline. |

**Figure S4** below illustrates with screenshots the main functionality of the PREHAB personal health system.


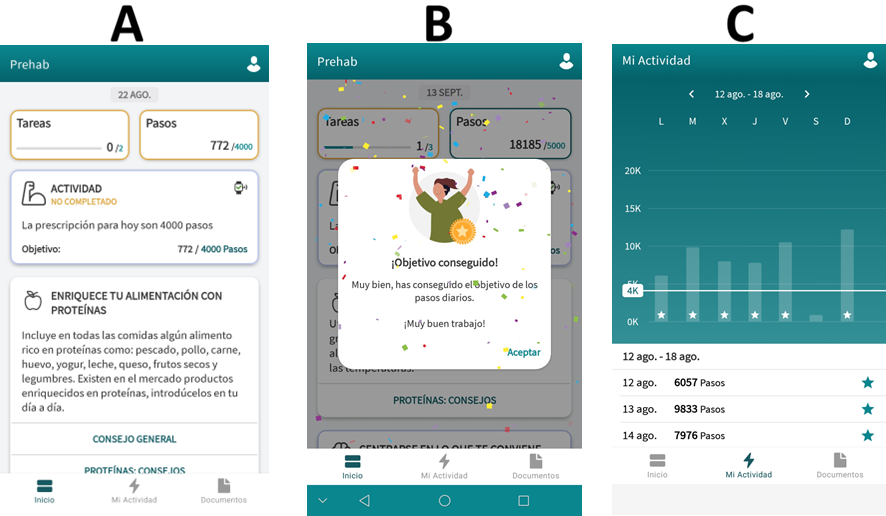


*Figure S4 – Look-and-feel of main functionalities of PREHAB personal health system: Home screen (Panel A) daily physical activity goal and nutrition and/or physical activity tips and mindfulness exercises. Gamification to enhance patient’s adherence to the program (Panel B). Summary of weekly physical activity (Panel C).*

Finally, the PREHAB personal health system (XCare) was integrated with SAP via ORM messaging triggered by the eventual prescription of Prehabilitation during the patient visit with the anaesthetist (**Figure S5**). The ORM message was securely bypassed between SAP and XCare with a Fast Healthcare Interoperable Resource platform (HAPI FHIR) deployed in the intranet of hospital information systems. Such bypass consisted on sending an invitation letter to the e-mail of the patient (if already informed in the Hospital SAP) with instructions on how to access the app-based version of the PREHAB personal health system and how to setup, for the first time, their password. The following diagram summarises the systems’ integration:


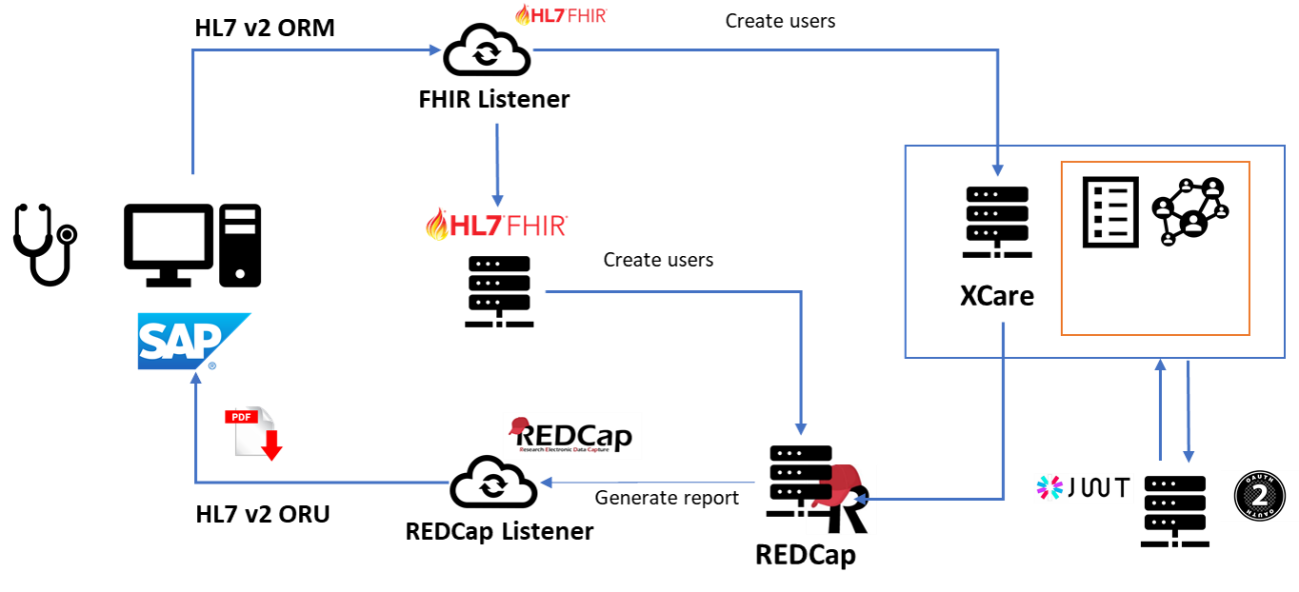
***Figure S5*** *– An interoperability middleware, using HL7 FHIR as common data model, was implemented to enable bidirectional interoperability among the Electronic Health Record (SAP), XCare system and REDCap electronic case report form.*

# Study III – Community-based care of chronic patients

**Objective:** Customize the CONNECARE Self-Management System (SMS - [www.connecare.eu](http://www.connecare.eu/)) to track adherence to a personalized daily physical activity prescription, with the remote support of a case manager (Study III).

As depicted in **Figure S6**, study III considered as key supporting technologies an adaptive case management platform to enhance collaborative work among health professionals (CONNECARE SACM) and patients themselves using a personal health system for patient self-management (CONNECARE SMS) at community level with off-line remote capture of patient reported outcomes (PROMs) and monitoring of daily physical activity (PA).


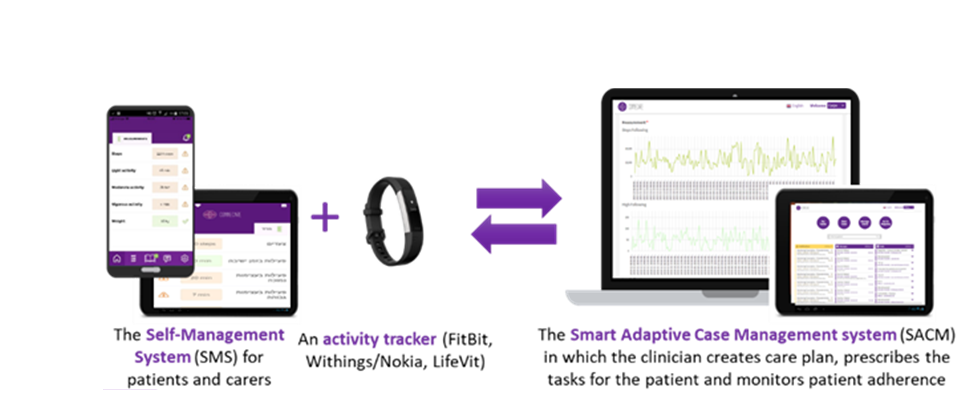


*Figure S6 – Look-and-feel of main functionalities of key supporting technology.*

For the design, technical deployment and integration of the supporting technologies in a stepwise manner, the PREHAB service prioritised the implementation of the personal health system for patient self-management at community level. Specifically, a personal health system, CONNECARE SMS, from EU project CONNECARE - Personalised Connected Care for Complex Chronic Patients, was adapted. CONNECARE SMS was responsible to give support to patients and carers for empowerment and engagement. It’s main functionalities are:

- Monitor physical activity (steps and level of activity) through API-based integrating LifeVit® pedometers.
- Manually add health measurements;
- Perform tasks and follow-up them: questionnaires; simple rehabilitation tasks, drugs intake and adherence;
- Consult advices automatically generated or sent by professionals and personalised for the given patient;
- Send/receive messages from the team of professionals in charge;
- Read and accept notifications;

*Table S3 – Adaptation requirements for CONNECARE SMS to support the prehabilitation service on Barcelona (Study I)*

| **Feature** | **Description** |
| --- | --- |
| **Spanish and Catalan languages** | Hospital Clínic facilitates translation to Spanish and Catalan both for the clinician’s portal (CONNECARE SACM) and the patient’s app (CONNECARE SMS) |
| **Monitoring of patient’s physical activity** | patient-specific target daily physical activity (i.e., number of daily steps) will be prescribed by healthcare professionals (number of target daily steps should be customisable dynamically and the prescription could be cancelled anytime.). Patients will receive physical activity prescriptions in CONNECARE SMS timeline (in the form of a daily goal), which can be manually answered or chosen to be directly linked via Bluetooth (requires integration of SDK) with a LiveVit pedometer (AT-250/AT-260) for automatic collection of daily steps. Patients will receive daily and weekly feedback (rewards, encroaching messages) with respect to the adherence to the physical activity goals. |
| **PROMs** | Spanish validated versions of the following questionnaires should be available for allocation at patient discharge:   - **YALE** - **HAD** - Patient Satisfaction questionnaire of the prehabilitation unit |

# Study IV – Enhanced management of frail chronic patients

**Objective:** To evaluate the potential of a digital health tool, Health-Circuit ([www.healthcircuit.es](http://www.healthcircuit.es)), for improving the management of complex chronic patients with risk of hospitalization, as well as the operational capacity of health teams in the community.

Health-Circuit allows healthcare professionals to easily adapt and customise shared care pathways over time, facilitating a “connected experience” for both the patient and the healthcare professionals. A web-based editor facilitates the creation and customisation of content (educational material, data collection instruments, tips, push notifications, etc.) and shared processes (roles, tasks for healthcare professionals, tasks for patients (PRMs/PREMs), objectives, etc.), without requiring a tight integration with existing electronic medical records. A secure and agile multimedia communication channel is then used by: (i) professionals to coordinate care with a personalised case management approach based on dynamic shared care plans; and by (ii) patients / caregivers to participating in effective health coaching and self-management strategies. Finally, a professional’s dashboard allows to monitor and benchmark outcome variables and key performance indicators for continuous improvement aiming to consolidate high-value care services.

A prototype version of Health Circuit (**Figure S7**) was developed for Study IV purposes, building on top of an existing corporate communication channel (Circuit by Unify^©^ - [www.circuit.com](http://www.circuit.com)) with bilateral or group interactions between patients and healthcare professionals, as starting functionality. The prototype version of Health Circuit consisted of an app for patients compatible with Android and iOS devices and the use of Circuit by Unify^©^ as backend for healthcare professionals.

***
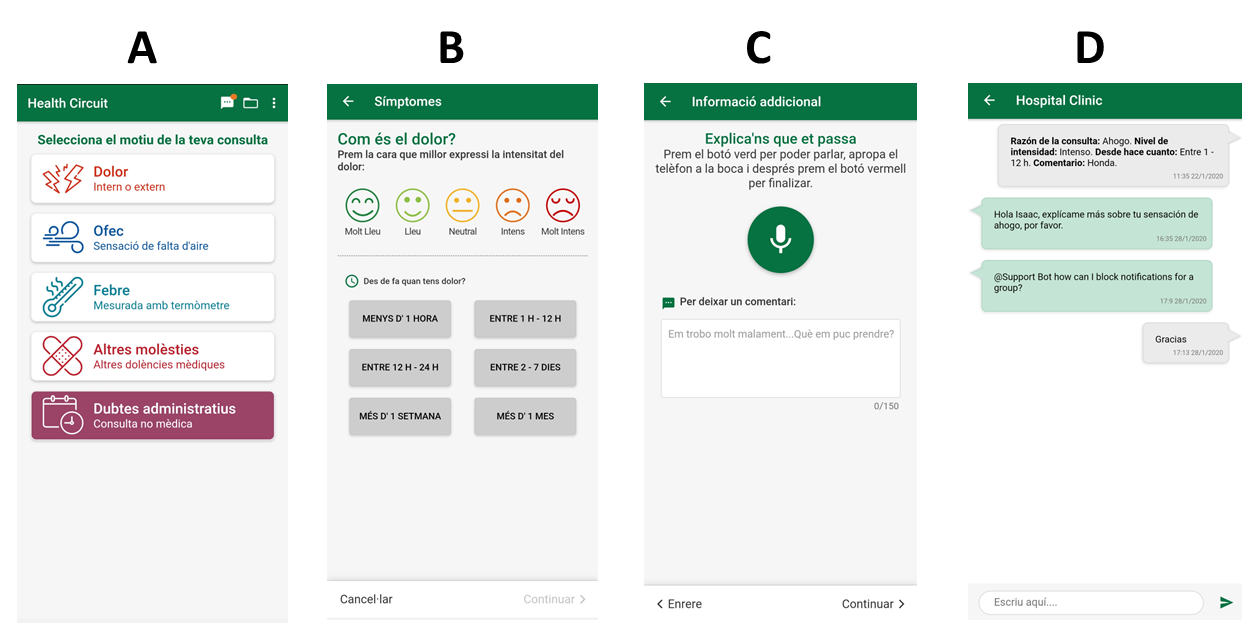
***

***Figure S7*** *– Main functionalities of Health Circuit for enabling patient-centric and cost-effective care management through innovative digital transformation with an integrated care approach. Home screen with main triage options for initiating a conversation with the case manager (Panel A). Information on intensity and duration of the reported health problem (Panel B). Text and/or audio recording for patient’s explanation about the reported health problem (Panel C). Resulting conversation with the case manager with the aim to tackle the health problem (Panel D).*

1. <https://eithealth.eu/product-service/paprika/> [↑](#footnote-ref-1)
