# Supplementary material for: The Assessment of Medical Device Software Supporting Health Care Services for Chronic Patients in a Tertiary Hospital: Overarching Study
Source: J Med Internet Res. 2023 Jan 4;25:e40976. doi: 10.2196/40976 (PMC9873251; doi:10.2196/40976)
Supplement: Multimedia Appendix 2 [file jmir_v25i1e40976_app2.docx]

# **Multimedia Appendix 2.** Details of the premarket analysis of the 4 pilot studies.

# Study I - Home-based non-invasive ventilation (NIV) of patients with hypercapnic respiratory failure.

*Technical performance*

2/3 of the technical problems that arose during the first three weeks of the study had a successful resolution. Most incidences had to do with the need to login with a username and password easy to forget.

*Patient experience*

The NPS was -3 (31% promoters, 34% passives and 34% detractors). However, the three Likert-scale questions rated from 1 (very bad) to 10 (very good) the general impression of the app (mean score of 7.5/10), its user friendliness (mean score of 8.2/10) and the ability to use the app without assistance (mean score of 8.5/10).

The mean patient usability score obtained was 78, which is considered as a good grading. Up to 42% of the participants used the link to the educational material and only 18% consulted the terms of use. Also, we found that 30% of the participants used the app through a family member or career.

*Professionals’ experience*

A dedicated nurse used a web-based portal to identify adherence problems. However, since the web-based portal was not the focus of study, its technical and usability performance was not assessed.

# Study II - Prehabilitation of high-risk patients undergoing major abdominal surgery

*Technical performance*

85% of the bugs reported during the pilot were either solved or an alternative solution was given. All issues were reported from users of Android devices. With respect to the type of observations during the pilot, 50% were due to comfortability and accessibility (system forces to use random password when the user resets it) and 30% to technology robustness.

*Patient experience*

The NPS was 31.25 (38% promoters, 56% passives and 6% detractors). The three Likert-scale questions rated from 1 (very bad) to 10 (very good) the general impression of the app (mean score of 8/10), its user friendliness (mean score of 8/10) and the ability to use the app without assistance (mean score of 7.5/10).

In the SUS, the mean patient usability score obtained was 67%, which is considered an average usability grading.

*Professionals’ experience*

The NPS score of the two physiotherapists that participated in the study was negative (-66.7). However, since the median of overall satisfaction is 5, we could consider that professionals had neutral experience using the web backend for professionals. This is in line with the reported mean SUS of 52%.

# Study III – Community-based care of frail chronic patients with the CONNECARE platform

*Technical performance*

75% of the bugs reported during the pilot were solved. Android devices were approximately reporting more bugs than iOS. Most of the observations during the pilot were due to lack of robustness of the Bluetooth connection with the pedometer (57%). Reported observations regarding motivation, reliability and comfortability and accessibility reached around 14% and 15%.

*Patient experience*

The NPS was -21 (37% promoters, 5% passives and 58% detractors). However, since the Likert-scale question with respect to the general impression of the app had a median of 6 out 10, we could consider that patients had a slightly positive experience using the CONNECARE system.

In the SUS, the mean patient usability score obtained was 56%, which is considered as slightly poor usability grading.

*Professionals’ experience*

During this pilot study, only one case manager was using the CONNECARE system. His NPS score was very low (1), so the professional involved would not recommend the CONNECARE system. This is in line with the rather poor perceived usability, with a SUS score of 42,5%.

# Study IV – Community-based care of frail chronic patients with Health Circuit

*Technical performance*

Most of the bugs reported during the pilot were either solved or an alternative solution was given. Only 2% of the bugs would not be solved. Android devices were approximately reporting 25% more bugs than iOS. Most of the observations during the pilot were due to usability (61.22%) and or comfortability and accessibility (32.65%) issues, mostly due to lack of robustness of the multimedia communication channel.

*Patient experience*

The NPS was 31.3 (44% promoters, 44% passives and 12% detractors). The same number of passive and promoters suggests that in general most patients had a positive experience using Health Circuit but not enough to be active promoters. This is reinforced by the fact that median of overall satisfaction is 7.8 out of 10.

Moreover, the mean patient usability score (SUS) obtained was 75.75%, which is considered above the average usability grading.

*Professionals’ experience*

The number of professionals who have had to use the Circuit platform is 7 (5 family doctors and 2 case managers). They had to intervene in a total of 15 events. The number of professionals per event has been maximum of 2. The case managers have intervened in all the events while the family doctors have participated in 8 of them.

The NPS score reported by professionals (n=5) is negative (-80). However, since the median of overall satisfaction is 5, we could consider that professionals had a neutral experience using Health Circuit. This is in line with the perceived usability, with a SUS score of 54%.
