# Supplementary material for: The Assessment of Medical Device Software Supporting Health Care Services for Chronic Patients in a Tertiary Hospital: Overarching Study
Source: J Med Internet Res. 2023 Jan 4;25:e40976. doi: 10.2196/40976 (PMC9873251; doi:10.2196/40976)
Supplement: Multimedia Appendix 3 [file jmir_v25i1e40976_app3.docx]

## **Multimedia Appendix 3.** Details of the postmarket surveillance analysis.

*Most prevalent malfunction issues*

In BfArM, we collected 4,258 whole medical events that included drugs, assays, and medical devices from 2017 to 2021, and 556 fitted the research requirements, with approximately 100 events being reported yearly (**Figure S1**).


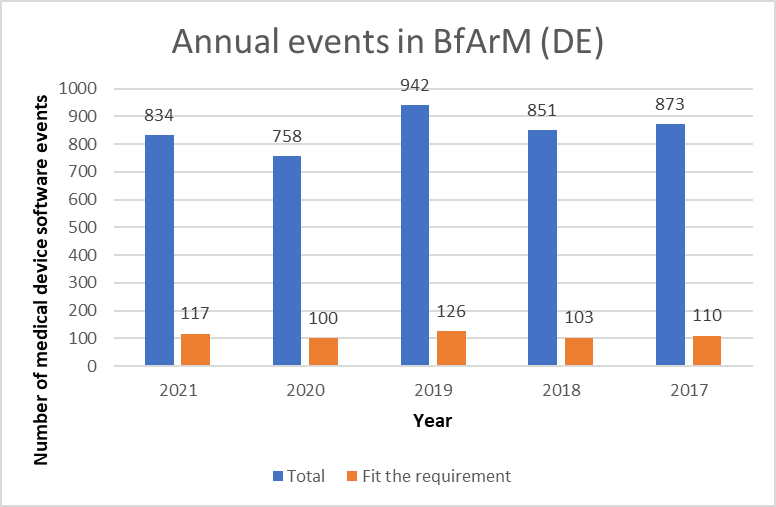


***Figure S1.*** *Number of annual medical events reported in BfArM database that met the research criteria from 2017 to 2021.*

‘Computer Software Problem’ was the most prevalent event with 293 cases between 2017 and 2021 (**Figure S2**). Some medical devices could not operate because of the ‘software problem ’, however the reports did not describe detail about the specific ‘software problem’.


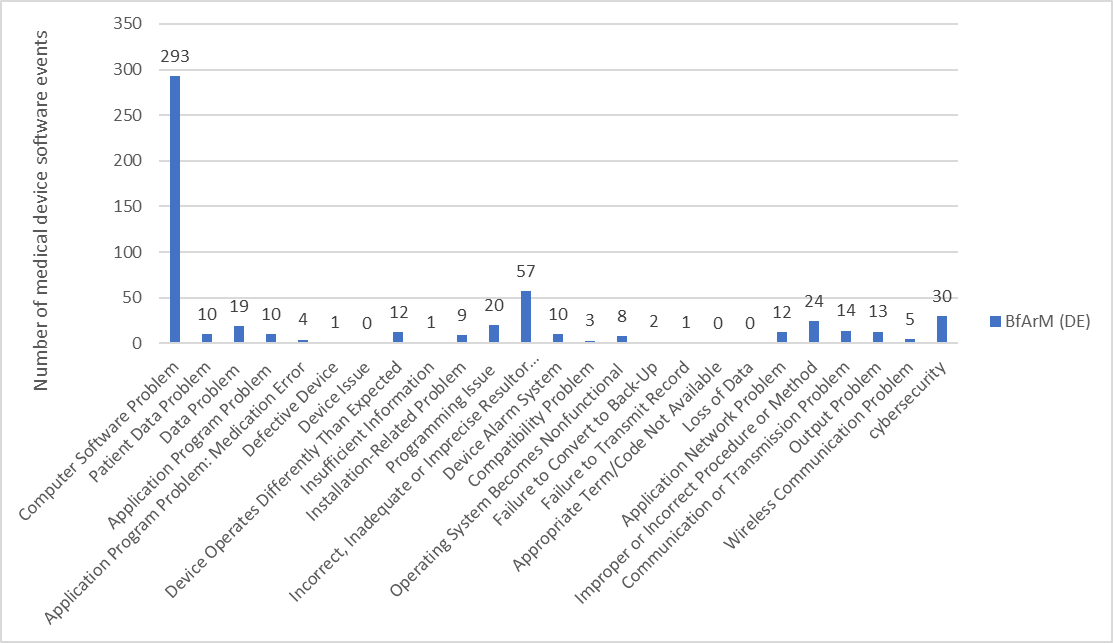


***Figure S2****. Number (vertical axis) of Malfunction factors (horizontal axis) of MDSW that meet the research criteria in the BfArM (DE).*

The second most reported issue was ‘Incorrect, Inadequate or Imprecise Result or Readings,’ it had 57 cases recorded on the BfArM database. Most of the ‘Incorrect, Inadequate or Imprecise Resultor Readings’ cases were caused by overdose calculations in X-ray, MRI, and in vitro diagnostic analyzers that induce the potential risk of harming health care providers and/or patients.

The third most prevalent issue was ‘cybersecurity’, with 30 cases from 2017 to 2021. Most of the reports described privacy & security vulnerabilities.

Other commonly reported issues were ‘Improper or Incorrect Procedure or Method’ and ‘Programming Issue’, such as an error in the procedure of the calculation or code error while users are operating the MDSW.

In the results of the MAUDE database, we found that, from 2017 to 2021, 638 events were related to MDSW, from which 114 events fit the research criteria (**Figure S3**).


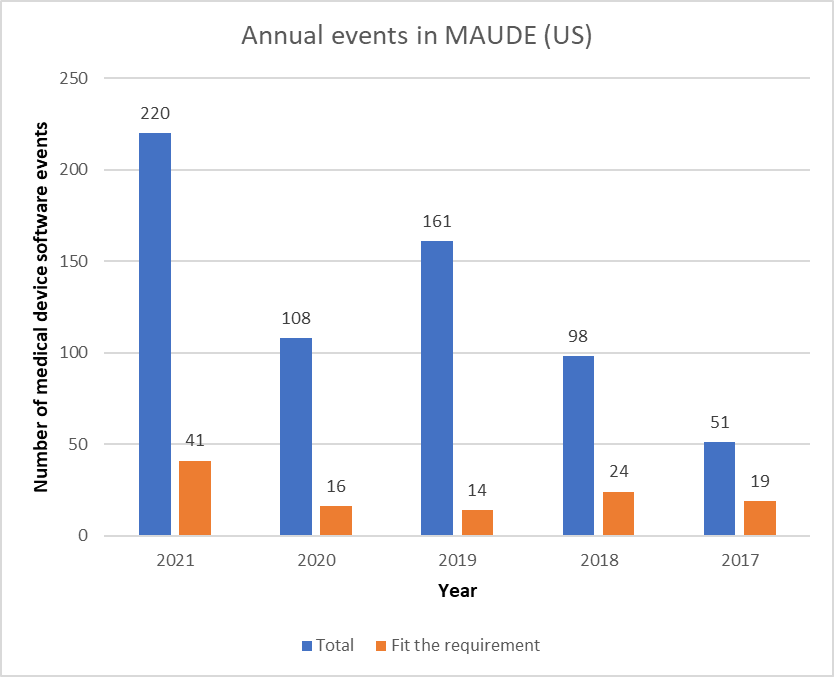


***Figure S3.*** *Number of annual medical events reported in MAUDE database that met the research criteria from 2017 to 2021.*

8 out of the total 114 events were reported as ‘Potential for patient harm’ and ‘Potential harm to a health care provider’. This is events were related to ‘Infusion safety management software’, ‘Index-Generating Electroencephalograph Software’, ‘Electronic Medical Record Software’, ‘Blood Establishment Computer Software And Accessories’, ‘Pharmacy Software System’, ‘Infusion safety management software’ and ‘software, transmission and storage, patient data.’

From all reported software medical device issues in MAUDE database, ‘Computer Software Problem’ was the most prevalent (**Figure S4**), such as the one that reported the battery cannot show the actual capacity on a screen, or users could not real-time view patient information on medical devices.

‘Application Program Problem’ was the second most commonly reported issue in MAUDE database. For example, a report detailed that an incorrect resuscitation icon may be displayed in a worklist resuscitation column when a resuscitation order type mapping is not complete in a responsive worklist view.


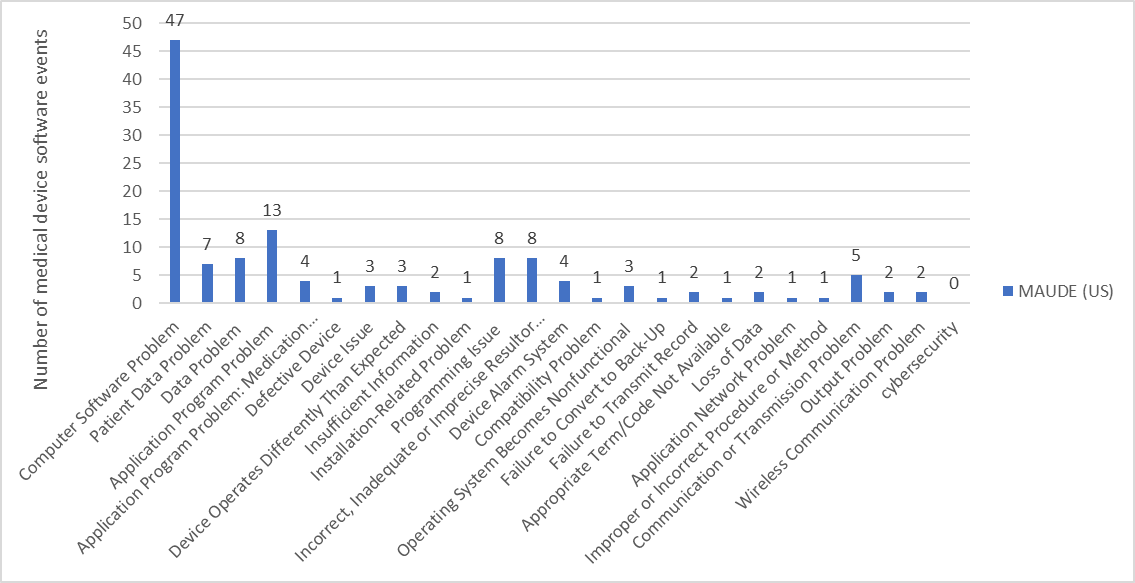


***Figure S4****. Number (vertical axis) of Malfunction factors (horizontal axis) of medical device software that meet the research criteria in the MAUDE database (US).*

‘Data Problem’ and ‘Programming Issue’ and ‘Incorrect, Inadequate or Imprecise Result or Readings’ were also commonly reported issues in MAUDE database.

*Thematic analysis results*

The thematic analysis based on all domains, except costs, for assessment of MDSW considered in the current study, resulted in the fact that most reported malfunction issues were related with technical Robustness (**Figure S5**), with almost all the 110 issues reported in the MAUDE database and 510 out of the 556 reported in the BfArM database. Therefore, most reported MDSW malfunction issues were technical software defects.

After technical robustness, ‘Privacy & Security malfunction issues were mostly reported in the German BfArM database (n=25), and finally, the usability domain was present in 17 malfunction issues reported in the BfArM database.

Neither other components of the technical domain, nor the clinical domain were present among the 556 and 114 malfunction issues reported in the BfArM and the MAUDE databases, correspondingly.


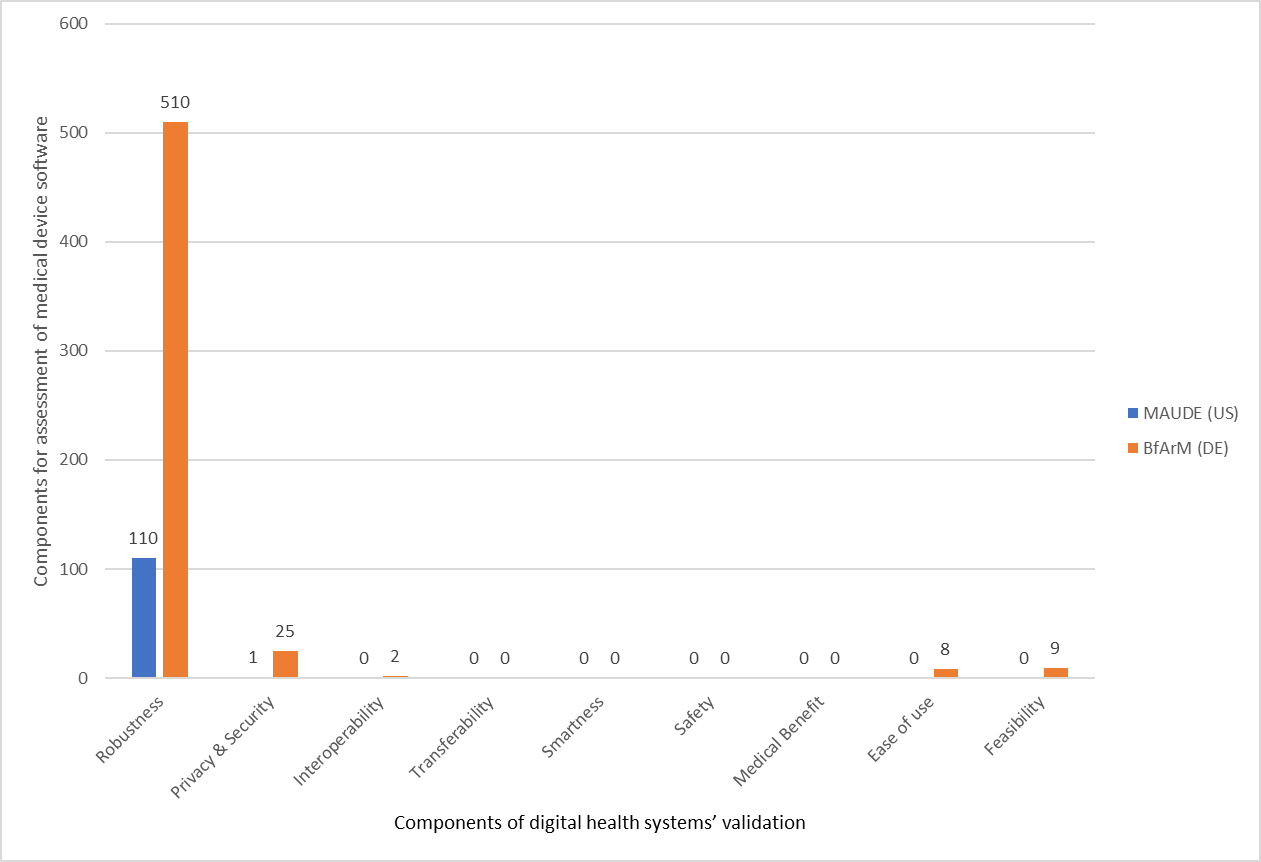


***Figure S5.*** *Result of the thematic analysis.*
